# Supplementary material for: Functional Mechanisms Underlying the Antimicrobial Activity of the Oryza sativa Trx-like Protein
Source: Int J Mol Sci. 2019 Mar 20;20(6):1413. doi: 10.3390/ijms20061413 (PMC6471494; doi:10.3390/ijms20061413)
Supplement: Supplementary file 1 [file ijms-20-01413-s001.pdf]

## Supplementary data

Figure S1.

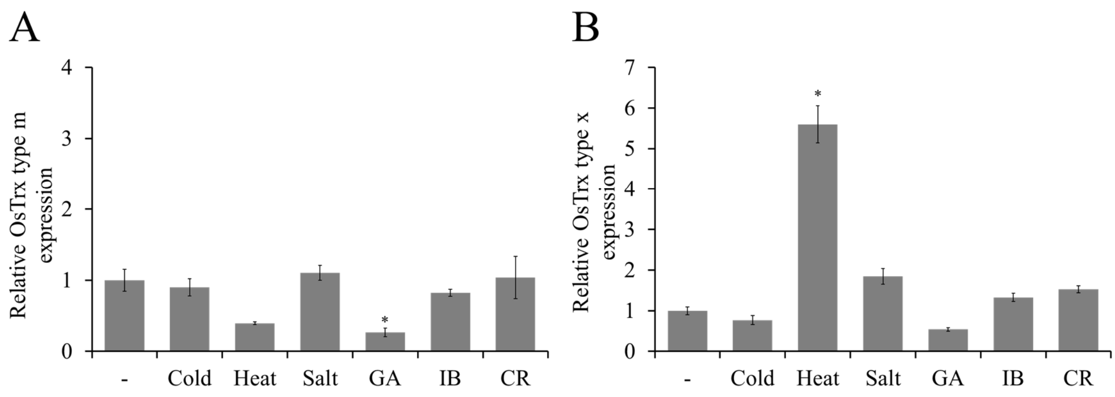


**Figure S1.** Expression level of two type *OsTrx* genes in rice treated with various external abiotic stresses. Rice seeds were irradiated with GA (200 Gy), IB (40 Gy), and CR (cosmic-rays). Ten-day-old rice seedlings were treated with heat (45°C), cold (4°C), and salt (200 mM NaCl). Error bars denote standard errors of biological replicates. Expression values of each gene are normalised against the expression of *OsActin*. Asterisks indicate statistical significance ($P < 0.05$, one-way ANOVA with a Tukey's *post hoc* test) of differences between control and stress treatment.

Figure S2.

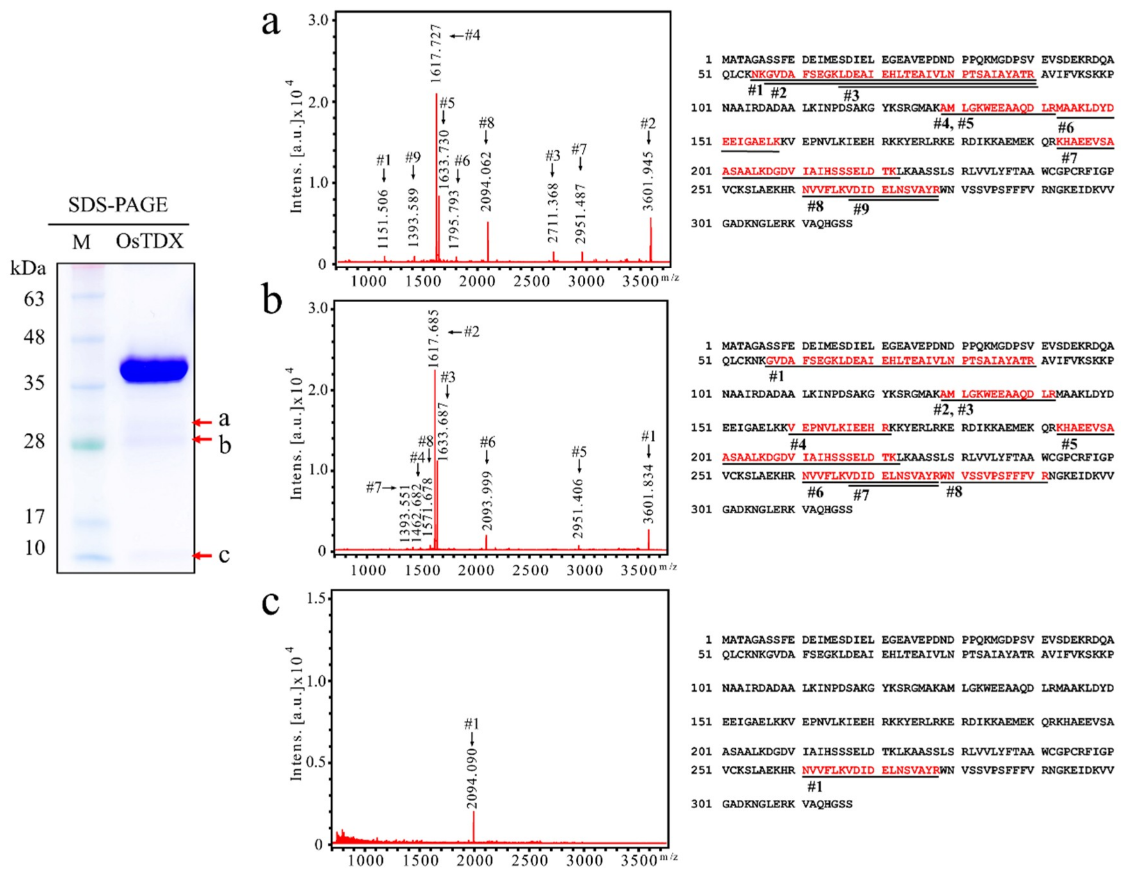


**Figure S2.** Purification and identification of OsTDX protein. Bacterially expressed recombinant OsTDX was resolved by 12 % SDS-PAGE (**left panel; Fig. 2A**) and MALDI-TOF analysis (**right panel**). (**a, b and c**) The labeled protein fragments by red arrows were identified as OsTDX proteins. Each trypsin digested peptide fragment (defined as #) is indicated by the underlined amino acids.
